# Absolute quantification of HTLV-1 basic leucine zipper factor (HBZ) protein and its plasma antibody in HTLV-1 infected individuals with different clinical status

**DOI:** 10.1186/s12977-016-0263-z

**Published:** 2016-04-27

**Authors:** Yasuo Shiohama, Tadasuke Naito, Toshio Matsuzaki, Reiko Tanaka, Takeaki Tomoyose, Hiroshi Takashima, Takuya Fukushima, Yuetsu Tanaka, Mineki Saito

**Affiliations:** Department of Microbiology, Kawasaki Medical School, 577 Matsushima, Kurashiki, Okayama 701-0192 Japan; Department of Neurology and Geriatrics, Kagoshima University Graduate School of Medical and Dental Sciences, 8-35-1 Sakuragaoka, Kagoshima, 890-8520 Japan; Department of Immunology, Graduate School of Medicine, University of the Ryukyus, 207 Uehara, Nishihara, Okinawa 903-0215 Japan; Division of Endocrinology, Diabetes and Metabolism, Hematology and Rheumatology, Second Department of Internal Medicine, Graduate School of Medicine, University of the Ryukyus, 207 Uehara, Nishihara, Okinawa 903-0215 Japan; Laboratory of Hematoimmnology, School of Health Sciences, Faculty of Medicine, University of the Ryukyus, 207 Uehara, Nishihara, Okinawa 903-0215 Japan; Division of Immunogenetics, Department of Immunobiology and Neuroscience, Medical Institute of Bioregulation, Kyushu University, Fukuoka, 812-8582 Japan

**Keywords:** HTLV-1, HBZ, ATL, Monoclonal antibody, ELISA

## Abstract

**Background:**

Human T cell leukemia virus type 1 (HTLV-1) basic leucine zipper factor (HBZ), which is encoded by a minus strand mRNA, is thought to play important roles in the development of adult T-cell leukemia (ATL) and HTLV-1-associated myelopathy/tropical spastic paraparesis (HAM/TSP). However, a comprehensive analysis of HBZ, including mRNA and protein expression, humoral immunoreactivity against HBZ, and HTLV-1 proviral load (PVL), in HTLV-1-infected individuals with different clinical status has not been reported previously.

**Results:**

In this study, using novel monoclonal antibody-based in-house enzyme-linked immunosorbent assay systems, we report the absolute quantification of HBZ protein and its plasma antibody in clinical samples from HTLV-1-infected individuals with different clinical status. The data were compared to both HBZ mRNA levels and PVL. The results showed that plasma anti-HBZ antibody was detectable only in 10.4 % (5/48) of asymptomatic carriers (ACs), 10.8 % (13/120) of HAM/TSP patients, and 16.7 % (7/42) of ATL patients. HBZ protein was detected in three out of five patients with acute ATL, but was not detected in patients with HAM/TSP (0/10) or ACs (0/4). Thus, an antibody response to HBZ was not associated with the PVL or the expression of HBZ (both at the mRNA and protein levels) or the clinical status of the infection.

**Conclusions:**

The present results emphasize the extremely low expression and immunogenicity of HBZ in natural HTLV-1 infection. However, there is a possibility that the low but distinct expression of HBZ protein in PBMCs is associated with the survival of HTLV-1-infected cells and the development of ATL.

**Electronic supplementary material:**

The online version of this article (doi:10.1186/s12977-016-0263-z) contains supplementary material, which is available to authorized users.

## Background

Human T cell leukemia virus type-1 (HTLV-1) has been linked to the development of adult T-cell leukemia (ATL) [1, 2] and a chronic inflammatory disease called HTLV-1-associated myelopathy/tropical spastic paraparesis (HAM/TSP) [3, 4]. HTLV-1 bZIP factor (HBZ) is a viral transcriptional regulator encoded in the minus strand of the 3ʹ-terminal region of the HTLV-1 genome [5]. HBZ is thought to play important roles in the pathogenesis of HTLV-1 associated diseases, because HBZ is the only viral gene that is genetically highly conserved [6], its mRNA is constitutively and ubiquitously expressed by all ATL cells and supports their proliferation [7], its mRNA expression is correlated with the HTLV-1 proviral load (PVL) and disease severity of HAM/TSP [8], its expression enhances the proliferative capacity of HTLV-1 infected T cells in culture and a transplantation model [9], and transgenic expression of HBZ in CD4+ T cells induced both T-cell lymphomas and systemic inflammation in mice [10]. The coding sequence of HBZ does not overlap with that of another viral transcription factor, Tax, which is also crucial for HTLV-1-mediated transformation, and HBZ acts in an manner opposite to Tax in the regulation of many viral and host genes [11, 12]. From an immunological perspective, HBZ differs significantly from other HTLV-1 proteins. A recent in silico analysis showed that strong binding of HBZ peptides [13], but not Tax peptides [which are immunodominant for circulating HTLV-1-specific cytotoxic T lymphocytes (CTLs)], to HLA class 1 molecules was associated with lower PVL and a reduced risk of developing HAM/TSP [13]. The predicted binding affinity of HLA to HBZ peptides was significantly weaker than that to the Tax peptides, and the detection frequency of HBZ-specific CTLs in HTLV-1-infected individuals was significantly lower than that of Tax-specific CTLs [14]. These observations suggest that efficient control of viral replication is associated with CTL recognition of poorly immunogenic HBZ protein, but not the immunodominant Tax protein [13], and that a strong immune response to HBZ is associated with a low PVL, which reduces the risk of both HAM/TSP [15] and ATL [16].

A previous in vitro study demonstrated that although high-avidity CTL lines against HBZ can be generated despite its poor immunogenicity [17], these CTL lines were able to lyse an autologous B-lymphoblastoid cell line (B-LCL) loaded with HBZ peptide, but not freshly isolated ATL cells [17]. More importantly, it was recently reported that recombinant vaccinia viruses expressing HBZ (rVV-HBZ) induce the production of CTLs in vivo that exert an anti-lymphoma effect in mouse and macaque models of ATL [18]. Specifically, vaccination with rVV-HBZ elicited specific T-cell responses in both mice and rhesus macaques. Furthermore, adoptive transfer of splenocytes obtained from rVV-HBZ-vaccinated mice significantly improved the survival of HBZ-expressing lymphoma cell-inoculated mice [18]. This study clearly demonstrates the importance of the HBZ protein and the potential of HBZ as a target antigen for immunotherapy of ATL [19]. Therefore, to develop preventive and therapeutic approaches for HTLV-1-associated diseases, it is important to examine the roles of HBZ and its immunological properties using clinical samples from HTLV-1-infected individuals with different clinical status. However, to date, there is no comprehensive analysis of HBZ with multiple parameters, including mRNA, protein, and anti-HBZ antibody levels and HTLV-1 PVL, in naturally infected cells from HTLV-1-infected individuals. In this study, we generated novel anti-HBZ monoclonal antibodies (mAbs) and developed an enzyme-linked immunosorbent assay (ELISA) to detect and quantify both HBZ protein and anti-HBZ antibody levels in clinical samples, to clarify the significance of HBZ in HTLV-1 infection.

## Methods

### Patients and cells

Peripheral blood was obtained from 120 patients with a clinical diagnosis of HAM/TSP, 42 ATL patients, 48 asymptomatic carriers (ACs), and 17 uninfected normal controls (NCs). The clinical profiles of the HTLV-1-infected individuals that participated in this study are shown in Table 1. Diagnosis of HAM/TSP and ATL was based on the World Health Organization diagnostic criteria [20] and Shimoyama criteria [21], respectively. This study was approved by the Research Ethics Committee of Kawasaki Medical School under the approval number of 1422-2, and written informed consent was obtained from all study subjects. Fresh peripheral blood mononuclear cells (PBMCs) were isolated by Histopaque-1077 (Sigma, St. Louis, MO) density gradient centrifugation, washed twice in RPMI 1640 with 10 % fetal calf serum (FCS), and stored in liquid nitrogen as stock lymphocytes until use. Plasma samples were stored at −80 °C until use. Seven HTLV-1-infected T-cell lines (C5/MJ, HUT-102, MT-1, MT-2, MT-4, SLB-1, TLOm1) and three HTLV-1-uninfected T-cell lines (CEM, Jurkat, MOLT-4) were used in this study. These cells were cultured in RPMI 1640 medium supplemented with 10 % heat inactivated FCS, 50 U/mL penicillin, and 50 µg/mL streptomycin (Wako, Osaka, Japan) at 37 °C in 5 % CO_2_.

**Table 1:** Clinical profiles of HTLV-1 infected individuals participated in this study

	ACs (n = 48)	HAM/TSP (n = 120)	ATL (n = 42)
Age	51.7 ± 18.3	61.4 ± 12.8	65.0 ± 13.5
Sex [n (%)]			
Male	17 (35.4)	34 (28.3)	21 (50.0)
Female	31 (64.6)	86 (71.7)	21 (50.0)
HTLV-1^a^ proviral load (median)	713.8 ± 1300.0 (323.0)	865.6 ± 883.4 (620.0)	7359.5 ± 5705.2 (6710.5)

The results represent the mean ± SD

^a^HTLV-1 Tax copy number per 10^4^ PBMCs

### Production of mAbs

To produce anti-HBZ mAbs, C57BL/6 mice or WKAH rats were immunized three or four times at 2-week intervals with keyhole limpet hemocyanin (KLH)-conjugated peptide fragments of HBZ (i.e., Peptides #1, #2, and #3; Additional file 1: Figure S1A) or histidine-tagged recombinant HBZ, which was produced in a wheat germ extract-based cell-free transcription and translation system (WEPRO7240H Expression kit; CellFree Sciences, Japan) (Additional file 1: Figure S1B). It is important to note that hbz gene is the only HTLV-1 gene with no nonsense mutation and it is genetically highly conserved among isolates, since human APOBEC3G generates nonsense mutations in the plus-strand coding sequence of HTLV-1 proviral genomes in vivo, targeting the minus strand of HTLV-1 during reverse transcription [6]. After the booster immunization, mouse or rat spleen cells were isolated and fused with mouse myeloma cells (Sp2/0-Ag14) using 50 % polyethylene glycol 2000 (Merck, Darmstadt, Germany). After selection in hypoxanthine–aminopterin–thymidine (HAT) medium, cells were cloned by limiting dilution (1 cell per well) with BM Condimed H1 (Roche, Indianapolis, IN), at least twice to ensure monoclonality. Individual clones growing in particular wells were tested by ELISA with the immunizing antigens.

### Transient transfection

The 293T cells were maintained in Dulbecco’s modified Eagle’s medium (Sigma, St Louis, MO) containing 10 % heat-inactivated FCS with 100 U/mL penicillin, 100 μg/mL streptomycin sulfate, and 2 mM l-glutamine. The HBZ expression plasmids were constructed as follows. First, the spliced isoform of HBZ, which is the most abundant HBZ transcript and significantly contributes to HBZ protein synthesis [22–24], was amplified from cDNA derived from the PBMCs of HAM/TSP patients by RT-PCR using the following primers: HBZ-F, 5ʹ-ATG GCG GCC TCA GGG CTG-3ʹ and HBZ-R, 5ʹ-TTA TTG CAA CCA CAT CGC CTC-3ʹ. Then, the amplified fragment was cloned into the pCMV-HA mammalian expression vector containing an N-terminal hemagglutinin (HA) epitope tag (Clontech Laboratories, Inc., Mountain View, CA). The 1 µg of pCMV-HA-HBZ vector was transiently transfected into 5.0 × 10^5^ 293T cells by Lipofectamine LTX with PLUS Reagent (Invitrogen, Carlsbad, CA) according to the manufacturer’s protocol. The empty pCMV-HA vector (1 µg) was used as a mock-transfected control.

### Indirect immunofluorescence

Cells were washed three times with phosphate-buffered saline (PBS) and fixed in 100 % ethanol for 5 min at −20 °C. Fixed cells were washed with wash buffer (PBS containing 0.1 % sodium azide and 0.1 % BSA), and then incubated with anti-HBZ mAbs for 20 min at 4 °C. After washing with wash buffer, cells were incubated with an Alexa Fluor 488-conjugated secondary Ab (goat anti-mouse IgG or goat anti-rat IgG; Cell Signaling Technology). In parallel, the nuclei in the cells were also stained blue with 4′,6-diamidino-2-phenylindole (DAPI). Then, the cells were washed with PBS and mounted with 20 % glycerol (Merck). Slides were examined with a LSM700 scanning laser confocal microscope (Carl Zeiss, Obercochen, Germany).

### Flow cytometry

Cells were fixed and stained as described in the indirect immunofluorescence section. After incubation with secondary mAbs, the cells were washed and resuspended in wash buffer and analyzed by flow cytometry (FACSCanto II; BD Bioscience, San Jose, CA) with FlowJo software (Tomy Digital Biology, Tokyo, Japan).

### Cell lysate preparation

Total proteins were extracted from uncultivated PBMCs or human T cell lines using Pierce RIPA Buffer (Thermo Fisher Scientific, Yokohama, Japan) with protease inhibitor cocktail (Thermo Fisher Scientific). Briefly, 1 × 10^7^ HTLV-1-infected and uninfected cells were washed three times with PBS, resuspended in Pierce RIPA Buffer with protease inhibitor cocktail, and then sonicated on ice using a Bioruptor^®^ sonicator (Diagenode, Liège, Belgium) according to the manufacturer’s protocol. After centrifugation (14,000×*g*, 4 °C, 15 min), the supernatants were collected for western blotting and sandwich ELISA.

### Western blotting

Whole-cell lysates from HTLV-1-infected and uninfected cells were subjected to SDS-polyacrylamide gel electrophoresis (SDS-PAGE) and transferred to polyvinylidene difluoride (PVDF) membranes (ATTO, Tokyo, Japan). PVDF membranes were blocked with 5 % skim milk in Tris-buffered saline containing 0.05 % Tween 20 (TBS-T) and probed with anti-HBZ mAbs or anti-Histone H3 mAb (Cell Signaling Technology, Danvers, MA). PVDF membranes were washed with TBS-T and incubated with horseradish peroxidase-conjugated secondary Abs (anti-rat IgG, HRP-linked whole Ab goat [NA935] or anti-mouse IgG, HRP-linked whole Ab sheep [NA931]; GE Healthcare, Buckinghamshire, England). After washing with TBS-T, the proteins were detected using ImmunoStar LD (Wako) and visualized with a C-DiGit^®^ Blot Scanner (LI-COR Biosciences, Lincoln, NE). To enhance the western blotting signals, Western BLoT Immuno Booster (Takara Bio, Shiga, Japan) was used according to the manufacturer’s protocol.

### Immunoprecipitation

The 293T cells in a 60 mm-diameter dish at 50 % confluence (6 × 10^5^ cells) were transfected with 3 μg of each indicated plasmids (i.e., pCMV-HA-HBZ or pCMV-HA-empty) using Lipofectamine LTX (Thermo Fisher Scientific, Waltham, MA) and then cultured for 24 h at 37 °C. The harvested cells were solubilized using lysis buffer (20 mM Tris–HCl [pH 7.9], 100 mM NaCl, 0.1 % Triton X-100). After sonication, homogenates were centrifugated at 14,000×*g* at 4 °C for 5 min, and the supernatant fraction was used as extracts for assay. Then HA-tagged HBZ proteins were isolated by immunoprecipitation with each indicated anti-HBZ monoclonal antibody. Namely, monoclonal antibody was mixed and rotated at 4 °C for 2 h with Protein A-Agarose Fast Flow (Sigma). The agarose beads were washed two times with lysis buffer to remove unbound antibodies. Then, cell extracts were mixed and rotated at 4 °C for 2 h. The agarose beads were washed two times with lysis buffer, and immunoprecipitated proteins were separated by 12 % SDS-PAGE, followed by western blotting using anti-HA monoclonal antibody (Roche, 3F10).

### Determination of HBZ-specific antibody titers in the plasma and cerebrospinal fluid (CSF) of HTLV-1-infected individuals

HBZ-specific antibody titers in the plasma of HTLV-1-infected individuals were determined by ELISA using a recombinant HBZ protein. Briefly, a 96-well flat-bottom plate (MaxiSorp; Nunc, Roskilde, Denmark) was coated with 50 µL of 1 µg/mL HBZ recombinant protein for 1 h at room temperature. Then, the wells were blocked with 1 % skim milk in PBS with 0.05 % Tween 20 (PBS-T) at room temperature for 1 h. After washing three times with PBS-T, 50 µL of 1:100 diluted human plasma or undiluted CSF samples was added to each well, and the plate was incubated for 1 h at room temperature. We selected 1:100 dilution of plasma in this ELISA, which showed the best performance (i.e., low background and high signal). After washing three times with PBS-T, 50 µL of HRP-conjugated goat anti-Human IgG F(ab′)_2_ (Jackson Immuno Research, West Grove, PA) was added to the wells, and the plate was incubated for 1 h at room temperature. After washing five times with PBS-T, 50 µL of SureBlue™ TMB Microwell Peroxidase Substrate (Kirkegaard & Perry Laboratories, Gaithersburg, MD) was added to the wells, and the plate was incubated for 5 min at room temperature. The development reaction was stopped with 50 µL of 2 M sulfuric acid (H_2_SO_4_), and then plates were read at 450 nm using a reference wavelength of 620 nm, with an automatic microplate reader (Multiskan™ FC; Thermo Fisher Scientific). Each sample was measured twice, and the results were determined by calculating the mean optical density (OD) for duplicate wells of each plasma sample. A positive result was defined as any value higher than the mean plus twice the standard deviation (SD) of the 17 NC samples (mean + 2SD = cut-off).

### ELISA analysis of HBZ protein levels in PBMCs from HTLV-1-infected individuals

Whole-cell lysates were prepared as described in the *Cell lysate preparation* section. HBZ protein levels in cell lysates were evaluated by an in-house sandwich ELISA using mAbs against HBZ (i.e., clone #91-1 for capture and clone #20-H12 for detection) (Additional file 1: Figure S2). Briefly, a 96-well flat-bottom plate (Nunc) was coated with an anti-HBZ mAb (clone #91-1: rat IgG1 mAb raised against peptide #3) at 4 °C overnight, and then blocked with 5 % skim milk in PBS-T containing 0.05 % azide and 0.05 % ProClin 30 (SIGMA) at room temperature for 30 min, followed by three washes with PBS-T. Recombinant HBZ was used as a standard and was diluted to a working concentration of 4000 ng/mL in antigen dilution buffer (PBS-T containing 0.1 % BSA, 0.05 % azide, and 0.05 % ProClin 30). Subsequently, serial twofold dilutions of the standard were made to generate a series with concentrations ranging from 4 to 4000 ng/mL. Then, 50 μL of diluted standard or samples (i.e., 50 µg total cell lysates which is equal to the amount used in western blotting) was added to the plate, and the plate was incubated for 1 h at room temperature. After washing the plate three times, 50 µL of diluted (0.2 µg/µL) HRP-conjugated anti-HBZ mAb (clone #20-H12: mouse IgG1 mAb raised against peptide #2) was added as a detection antibody, and the plate was incubated for 1 h at room temperature along with solution-1 of Western BLoT Immuno Booster (Takara Bio). After washing the plates five times, 100 μL of TMB 1-Component Microwell Peroxidase Substrate, SureBlue (Kirkegaard & Perry Laboratories) was added to each well, and the plate was incubated for 5 min at room temperature. The development reaction was stopped with 50 µL of 2 M H_2_SO_4_, and then the plates were read at 450 nm using a reference wavelength of 620 nm, with an automatic microplate reader (Multiskan FC). The cut-off value for the HBZ sandwich ELISA was calculated as the mean OD reading plus twice the standard deviation of four HTLV-1 uninfected cells (i.e., CEM, Jurkat, and two normal uninfected PBMCs). HRP was conjugated to the mAb using the Peroxidase labeling kit (DOJINDO, Kumamoto, Japan) according to the manufacturer’s protocol.

### Genomic DNA and RNA extraction and cDNA synthesis

Genomic DNA was extracted from PBMCs using the QIAamp blood kit (QIAGEN, Tokyo, Japan). RNA was extracted from PBMCs using the RNeasy Mini Kit with on-column DNase digestion (QIAGEN, Hilden, Germany). Complementary DNA (cDNA) was synthesized using the PrimeScript^®^ RT reagent Kit (Takara Bio). All procedures were performed as suggested by the manufacturer.

### Quantification of HTLV-1 PVL

To measure the HTLV-1 PVL in PBMCs, we used a quantitative PCR method with 100 ng of genomic DNA (roughly equivalent to 10^4^ PBMCs) and the ABI 7500 Fast Real-Time PCR System (Applied Biosystems, Foster City, CA). We used β-actin gene for normalization of PVL per cell. Namely, since single PBMC has two copies of β-actin, the HTLV-1 PVL was determined using the following formula: HTLV-1 *tax* copy number per 1 × 10^4^ PBMCs = [(*tax* copy number)/(β-actin copy number/2)] × 10^4^. Further details of this mothod are given in previous work [15]. All samples were analyzed in triplicate.

### Real-time RT-PCR analysis

We performed TaqMan^®^ real-time RT-PCR assays to determine the quantitative differences in the expression of HBZ mRNA as previously reported [8]. In real-time RT-PCR analysis, we detected the spliced isoform of HBZ, which is the most abundant HBZ transcript and significantly contributes to HBZ protein synthesis [22–24]. HBZ mRNA expression were normalized to the expression of the hypoxanthine ribosyl transferase (HPRT) gene (Human HPRT1 Endogenous Control 4333768; Applied Biosystems). Namely, serially diluted cDNA from HTLV-1 infected MT-2 cells was used for generating standard curves for the value of HBZ mRNA and HPRT mRNA, and the relative HBZ mRNA load was calculated by the following formula: HBZ mRNA load = value of HBZ/value of HPRT. Further details of this mothod are given in previous work [8].

### Statistical analysis

The Mann–Whitney U test was used to compare data between two groups. Correlations between variables were examined using Spearman’s rank correlation analysis. The results shown are the mean ± SD where applicable. P values less than 0.05 were considered statistically significant.

## Results

### Development of mAbs against HBZ

**Fig. 1:**
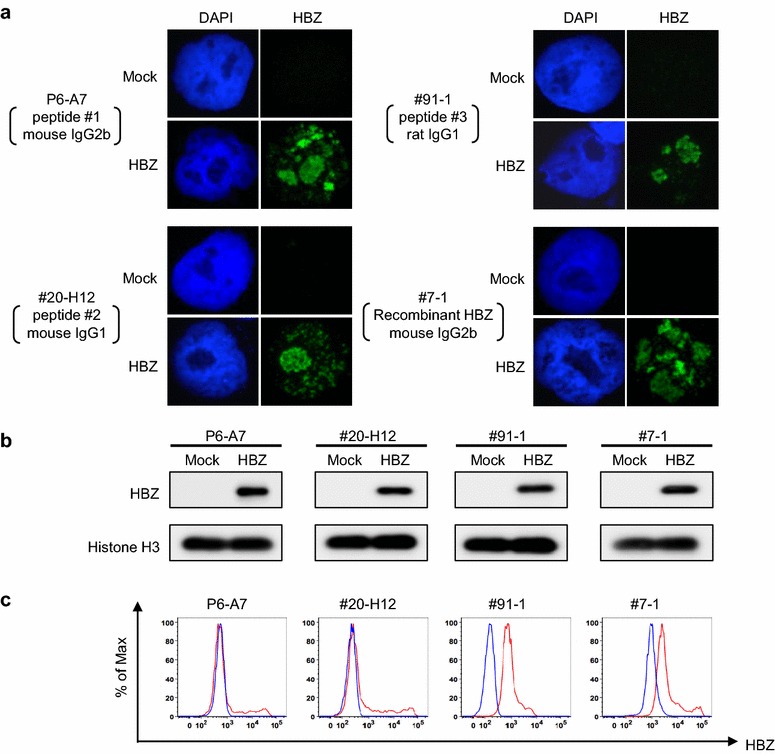
Generation and characterization of anti-HTLV-1 bZIP factor (HBZ) monoclonal antibodies (mAbs). **a** HBZ protein levels in HBZ-transfected 293T cells were analyzed by indirect immunofluorescence using anti-HBZ mAbs P6-A7 (raised against peptide #1, mouse IgG2b), #20-H12 (raised against peptide #2, mouse IgG1), #91-1 (raised against peptide #3, rat IgG1), and #7-1 (raised against recombinant HBZ, mouse IgG2b). **b** HBZ expression vector-transfected 293T cells (HBZ) and mock-transfected 293T cells (Mock) were analyzed by western blotting using the novel anti-HBZ mAbs. **c** HBZ expression vector-transfected 293T cells (*red line*) and mock-transfected 293T cells (*blue line*) were analyzed by flow cytometry using the novel anti-HBZ mAbs. Three independent experiments were performed, and representative data are shown in *each figure*

We established seven hybridoma clones secreting HBZ-reactive IgG antibodies (Additional file 2: Table S1). These antibodies were examined for antigen specificity, HBZ expression, and HBZ localization after purification. Antibody reactivity and specificity were confirmed by the detection of HBZ protein in the cell extracts of 293T cells transfected with the HBZ expression plasmid pCMV-HA-HBZ, but not in extracts from mock-transfected cells (transfected with pCMV-HA-Mock; Fig. 1). As previously reported [5], indirect immunofluorescence of 293T cells transfected with an HBZ expression plasmid showed a granular distribution of HBZ protein in the nucleus (Fig. 1a). Western blotting of 293T cells transfected with the HBZ expression plasmid showed a single prominent band of the predicted length for full-length HBZ protein (31 kDa), whereas mock-transfected 293T cells did not show any bands (Fig. 1b). We also attempted to detect HBZ protein by flow cytometry (Fig. 1c). As shown in Fig. 1c, 293T cells transfected with the HBZ expression plasmid were specifically stained with the anti-HBZ mAbs, whereas mock-transfected cells were not. These results confirm the specificity of our mAbs against HBZ protein, and demonstrate that they can be used for intracellular flow cytometric analysis.

### The expression of endogenous HBZ protein in HTLV-1-infected cell lines

**Fig. 2:**
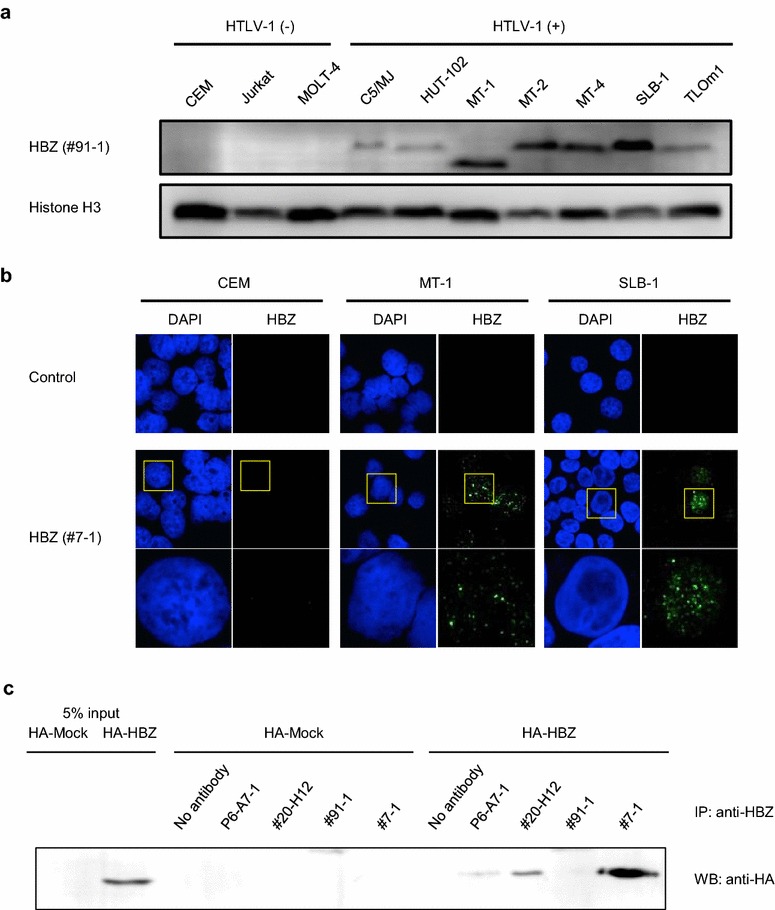
HBZ protein expression in HTLV-1-infected cell lines. **a** HBZ protein expression in HTLV-1-infected cell lines was analyzed by western blotting using anti-HBZ mAb #91-1 (raised against peptide #3, rat IgG1), which gave highest sensitivity among four mAbs (i.e. P6-A7, #20-H12, #91-1, #7-1). Histone H3 was used as a loading control for the nuclear fraction. **b** HBZ protein expression in HTLV-1-infected cell lines (MT-1 and SLB1) was analyzed by immunofluorescence microscopy using anti-HBZ mAb #7-1 (raised against recombinant HBZ, mouse IgG2b), which gave highest sensitivity among four mAbs (i.e. P6-A7, #20-H12, #91-1, #7-1). Three independent experiments were performed, and representative data are shown in *each figure*. **c** Immunoprecipitation assay using four different anti-HBZ mAbs. P6-A7, #20-H12, and #7-1 were available for immunoprecipitation

To confirm the expression and localization of HBZ in chronically infected cells, we performed western blotting and indirect immunofluorescence staining of naturally HTLV-1-infected T cell lines (Fig. 2a, b). Among the four anti-HBZ monoclonal antibodies (i.e., P6-A7, #20-H12, #91-1, #7-1) tested, #91-1 and #7-1 antibodies are suitable for western blotting, and #91-1 provides the greatest sensitivity (Additional file 1: Figure S3). As shown in Fig. 2a, western blotting with #91-1 raised against peptide #3 (rat IgG1) showed that the HBZ protein was expressed in all the HTLV-1-infected T cell lines examined, but was not detected in non-infected T cell lines (Fig. 2a). Interestingly, in the HTLV-1-infected T cell line MT-1, the HBZ protein was a lower molecular weight than in the other infected cell lines. Since sequence analysis revealed that the coding sequence of the HBZ gene in MT-1 cells is completely conserved (data not shown), it is supposed that post transcriptional regulation of HBZ protein may be the cause of this observation. We also examined HBZ in the HTLV-1-infected T cell lines by immunofluorescence with our mAb raised against recombinant HBZ (clone #7-1, mouse IgG2b), which provides the greatest sensitivity (Fig. 2b). As shown, the HTLV-1-infected T cell lines MT-1 and SLB1 exhibited a granular nuclear distribution, as was observed in the 293T cells transfected with the HBZ expression plasmid, whereas the HTLV-1 uninfected T cell line CEM did not. We further investigated whether our in-house mAbs against HBZ is suitable for immunoprecipitation. As shown in Fig. 2c, among the four anti-HBZ mAbs tested (P6-A7, #20-H12, #91-1, #7-1), P6-A7, #20-H12, and #7-1 were available for immunoprecipitation.

### Detection of anti-HBZ antibodies in some plasma samples from HTLV-1-infected individuals with different clinical status

**Fig. 3:**
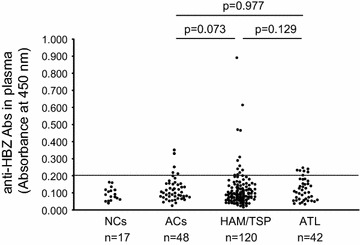
Detection of HBZ-specific antibodies in the plasma of HTLV-1-infected individuals with different clinical status. Antibodies against HBZ in plasma samples from HTLV-1-infected individuals were detected by ELISA using a recombinant HBZ protein as the coating antigen. Each plasma sample (50 µL of a 1:100 dilution) was examined twice, and the results shown are the mean optical density (OD) of duplicate wells. A positive result was any value higher than the mean OD plus twice the standard deviation (*dotted line* represents the cut-off value) of the 17 NC plasma samples. Differences between groups were analyzed with the Mann–Whitney U test

**Fig. 4:**
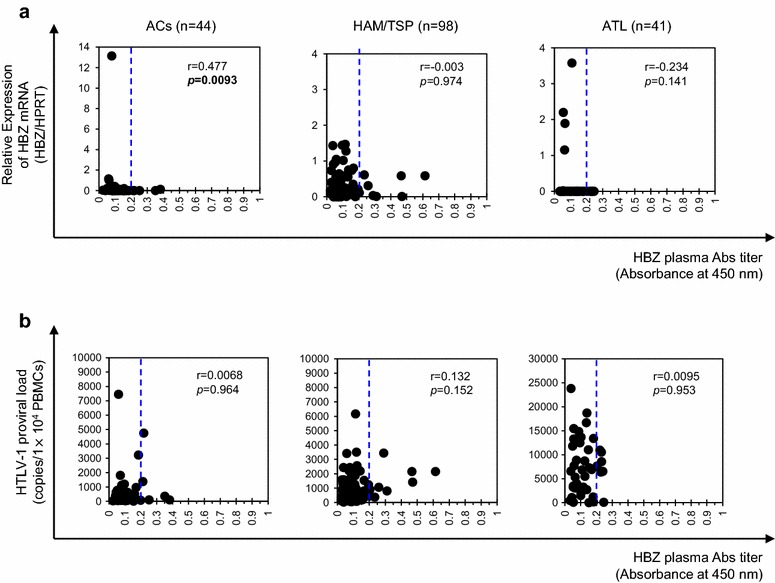
Correlation analysis between plasma anti-HBZ antibody levels and HTLV-1 mRNA levels or proviral load in HTLV-1-infected individuals. **a** HTLV-1 HBZ mRNA levels were significantly correlated with plasma anti-HBZ antibody levels in ACs (p = 0.0093, r = 0.477), but not in HAM/TSP patients (p = 0.974, r = −0.003) or ATL patients (p = 0.141, r = −0.234) by Spearman’s rank correlation analysis. **b** HTLV-1 HBZ mRNA levels were not significantly correlated with HTLV-1 PVL in ACs (p = 0.964, r = 0.0068), HAM/TSP patients (p = 0.152, r = 0.132), and ATL patients (p = 0.953, r = 0.0095). Correlations were examined by Spearman’s rank correlation analysis. The *dotted line* represents the cut-off, i.e. the mean OD plus twice the standard deviation of the 17 NC plasma samples

To investigate the relationship between the expression level of HBZ mRNA and the antibody response against HBZ in Japanese HTLV-1-infected individuals with different clinical status (i.e., HAM/TSP, ATL, and AC), we quantified the expression of HBZ mRNA in PBMCs and anti-HBZ Abs in plasma. First, using plasma samples from 210 HTLV-1-infected individuals (48 ACs, 42 patients with ATL, and 120 patients with HAM/TSP), we evaluated plasma anti-HBZ Abs by ELISA with a recombinant HBZ protein. These data were then compared to both HBZ mRNA expression and PVL. As shown in Fig. 3, immunoreactivity against HBZ was detected only in 10.4 % (5/48) of ACs, 10.8 % (13/120) of patients with HAM/TSP, and 16.7 % (7/42) of patients with ATL. Although a high titer of anti-HBZ antibody was detected in the plasma of certain patients with HAM/TSP, there was no statistically significant difference among the three groups. In these anti-HBZ antibody-positive individuals, the antibody response to HBZ did not correlate with HBZ mRNA expression, except in ACs (Fig. 4a), and the antibody response to HBZ did not correlate with the PVL in any of the test groups (Fig. 4b). This observation is consistent with a previous study, which also demonstrated that antibody responses against HBZ did not correlate with HBZ mRNA expression or PVL in patients with HAM/TSP [25]. Next, we examined the antibody responses against HBZ in CSF, since strong antibody responses against HTLV-1 antigens other than HBZ have been reported in the CSF of HAM/TSP patients [26, 27]. However, antibodies against HBZ were not detected in any of the CSF samples examined (0 out of 120, data not shown).

### Detection of HBZ proteins in the PBMCs of ATL patients but not in HAM/TSP patients or ACs

**Fig. 5:**
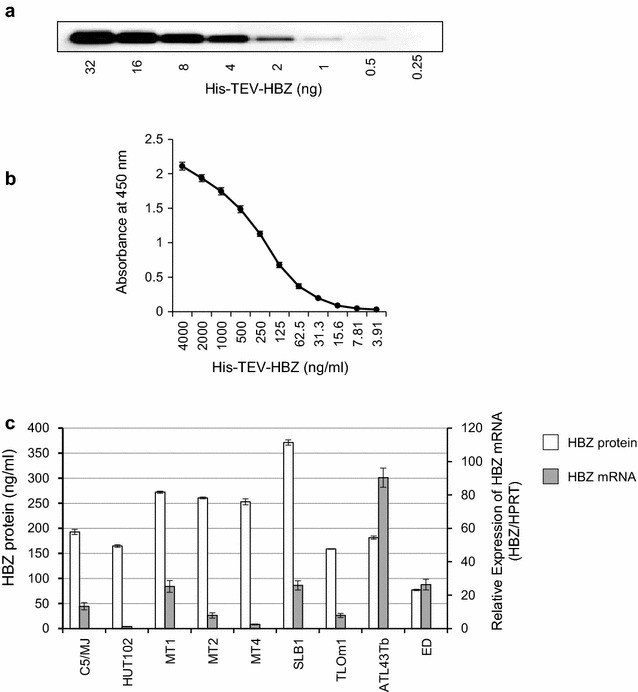
Quantification of HBZ protein expression levels in HTLV-1-infected T-cell lines. The absolute intracellular concentration of an endogenous HBZ protein in HTLV-1-infected T-cell lines was analyzed by a novel HBZ sandwich ELISA. **a** Western blot image of twofold serially diluted recombinant HBZ protein probed with anti-HBZ mAb (clone #91-1). **b** A representative standard curve for the ELISA comprised of twofold serial dilutions of recombinant HBZ protein (from 4000 to 3.91 ng/mL). **c** Absolute quantification of HBZ protein in HTLV-1-infected T-cell lines. Results are presented as mean ± SD of three independent experiments with duplicate wells

**Fig. 6:**
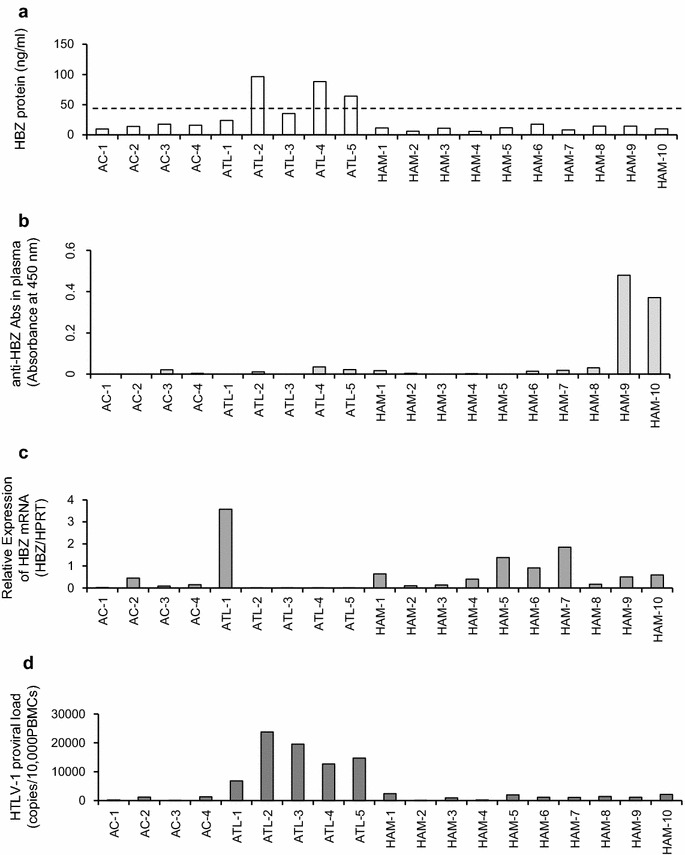
Quantification of HBZ protein expression levels and comparison to HBZ mRNA load, anti-HBZ antibody levels, and PVL in clinical samples from HTLV-1-infected individuals with different clinical status. **a** HBZ protein expression levels in naturally infected PBMCs of HTLV-1-infected individuals with different clinical status, evaluated by an in-house sandwich ELISA using mAbs against HBZ. HBZ protein was detected in three out of five acute ATL patients examined, but not in HAM/TSP patients (zero out of ten) or ACs (zero out of four). The *dotted line* represents the cutoff, i.e. the mean OD plus twice the standard deviation of four HTLV-1 uninfected cells (CEM, Jurkat, and two normal uninfected PBMCs). **b** Anti-HBZ antibody levels in plasma of HTLV-1-infected individuals with different clinical status determined by ELISA using a recombinant HBZ protein. **c** HBZ mRNA levels in PBMCs of HTLV-1-infected individuals with different clinical status determined by real-time PCR. HBZ mRNA expression were normalized to the expression of the hypoxanthine phosphoribosyltransferase gene (Human HPRT1 Endogenous Control 4333768; Applied Biosystems). **d** HTLV-1 proviral load (PVL) in PBMCs of HTLV-1-infected individuals with different clinical status. The HTLV-1 PVL was determined using the following formula: HTLV-1 *tax* copy number per 1 × 10^4^ PBMCs = [(*tax* copy number)/(β-actin copy number/2)] × 10^4^. All samples were analyzed in triplicate

To determine the absolute intracellular concentration of HBZ protein in naturally HTLV-1-infected cells, we developed a sandwich ELISA using our in-house anti-HBZ mAbs (Additional file 1: Figure S2). Recombinant HBZ was used as a standard. Western blot images of twofold serially diluted recombinant HBZ probed with an anti-HBZ mAb (clone #91-1) and a representative standard curve for the ELISA are shown in Fig. 5a, b, respectively. Using this system, we report the absolute quantification of HBZ protein in HTLV-1-infected cells. Although the levels of HBZ detected by ELISA match the levels of HBZ from western blotting when we quantify the intensity of HBZ band relative to Histone H3 in each sample by ImageJ software (data not shown), HBZ protein and mRNA levels were not correlated in HTLV-1-infected T cell lines (Fig. 5c). As shown in Fig. 5a, #91-1 can detect at least 1 ng of HBZ protein by western blotting. In the recent report by Raval et al. [28], their anti-HBZ mAb 4D4-F3 could detect at least 2 ng of purified GST-tagged HBZ protein, indicating nearly equivalent sensitivity to #91-1. We further examined the expression levels of HBZ protein in the PBMCs of HTLV-1-infected individuals with different clinical status, and the data were compared to both HBZ mRNA levels and PVL (Additional file 2: Table S2; Fig. 6). HBZ protein was detected in three out of five acute ATL patients examined, but was not detected in any HAM/TSP patients (zero out of ten) or ACs (zero out of four) (Fig. 6a). Further, the HBZ protein data were compared to anti-HBZ antibody levels in plasma (Fig. 6b), HBZ mRNA levels in PBMCs (Fig. 6c), and PVL levels in PBMCs (Fig. 6d). We observed that the amount of HBZ protein in primary HTLV-1-infected cells was correlated with PVL but not with either anti-HBZ antibody or HBZ mRNA levels.

## Discussion

HBZ has been reported to play important roles in the pathogenesis of both ATL and HAM/TSP. However, to date, there is no report of a comprehensive analysis of HBZ mRNA and protein expression, anti-HBZ Ab levels, and HTLV-1 PVL in patients with ATL and HAM/TSP as well as ACs. To further our understanding of HTLV-1-associated diseases and to aid in the development of new therapies, it is important to evaluate the expression profiles of HBZ and the host immune response against HBZ using samples from HTLV-1-infected patients with different clinical status. In particular, direct analysis of native, endogenous HBZ protein is crucial. In this study, we developed new mAbs specific for HBZ that can be used for flow cytometry, western blotting, ELISA, and immunofluorescence microscopy. We observed the expression and localization of native, endogenous HBZ protein in HTLV-1-infected human T cells. Our results are consistent with previous observations that HBZ protein was expressed in all HTLV-1-infected T cell lines examined and is localized to the nucleus, with a speckle-like distribution [28]. Similar results were observed in cells overexpressing HBZ protein [5, 23, 28–30], indicating that this subcellular localization of HBZ protein is not due to overexpression or non-physiological conditions. A recent report by Raval et al. showed that native HBZ protein in the nucleus co-localizes with many transcriptional regulators, such as p300, JunD, CBP, and CREB2 [28]. Molecular analyses, such as chromatin immunoprecipitation, may identify a large set of HBZ-targeted genes, which might be involved in the pathogenesis of HTLV-1-associated diseases. Since our mAbs P6-A7, #20-H12, and #7-1 were available for immunoprecipitation, these mAbs may be useful tools for future functional studies for HTLV-1 and its associated diseases.

Recently, two studies suggested the existence of anti-HBZ antibodies in HTLV-1-infected individuals [25, 31]. Enose-Akahata et al. reported the antibody responses against HBZ in US-resident patients with HAM/TSP of mainly African descent [25]. Furuta et al. found one blood donor with antibodies against HBZ among 114 HTLV-1-positive plasma samples from Japanese Red Cross blood centers (0.88 %) in Japan; these samples were assumed to be from ACs [31]. Using our HBZ-specific ELISA, we screened 210 plasma samples, including 48 from ACs, 120 from HAM/TSP patients, and 42 from ATL patients, for immunoreactivity against HBZ, and the results were compared to both the HBZ mRNA levels and the PVL in PBMCs of these HTLV-1-infected individuals. Anti-HBZ antibodies were detected in 10.4 % (5/48) of ACs, 10.8 % (13/120) of HAM/TSP, and 16.7 % (7/42) of ATL patients in our Japanese samples. These results are consistent with a previous US study showing that anti-HBZ antibody was detected in 10.34 % (15/145) of ACs, 13.46 % (14/104) of HAM/TSP patients, and 12.36 % (11/89) of ATL patients [25]. In addition, consistent with this US study, our data showed that the antibody responses against HBZ were not correlated with PVL or HBZ mRNA expression in HAM/TSP patients. We also examined the antibody responses against HBZ in the CSF of HAM/TSP patients. Although the previous study showed HBZ-specific antibody responses in the CSF in two out of five patients examined, albeit at lower levels than in serum, we did not detect antibodies against HBZ in any of the 120 CSF samples examined. We conclude that HBZ-specific antibody responses in CSF of HAM/TSP patients were significantly weaker than the response to other HTLV-1 antigens such as Gag, Env and Tax, because antibody responses against these molecules can be found in both serum and CSF; in certain patients the titers are higher in CSF than in plasma [25]. These findings indicate that the frequency of antibody responses to HBZ was surprisingly low compared to antibody responses to other viral proteins such as Gag, Env and Tax (each more than 90 %) [32, 33]. This low frequency is probably due to the low immunogenicity of HBZ or the low sensitivity of the method used. In any case, it is clear that antibody responses to HBZ can be detected in a proportion of individuals in vivo in the course of natural HTLV-1 infection, with similar frequencies in two ethnically diverse HTLV-1-infected populations (i.e. Japanese and American of mainly African descent), which carry different subgroups of HTLV-1 [25]. It is noteworthy that in human immunodeficiency virus (HIV) infection, antibody responses against the viral transcription factor Tat, which plays an important role in viral infectivity and pathogenicity, are also detected only in a small proportion of HIV-infected individuals (30 out of 252 subjects, 11.9 %) [34]. Since the presence of anti-Tat antibodies is associated with delayed disease progression [34, 35], it will be interesting to see whether the presence of anti-HBZ antibodies is associated with the clinical course of HTLV-1-associated diseases. However, we did not find any significant relationship between the presence of anti-HBZ antibodies and disease progression in HAM/TSP patients (data not shown). In addition, neither the previous study nor our current study showed a significant association between the frequency of a humoral immune response to HBZ and the clinical status of HTLV-1 infection (i.e., HAM/TSP, ACs, and ATL) [25]. In contrast, although the previous study showed a significant difference in the HBZ-specific antibody responses between ATL patients with chronic and lymphoma subtypes [25], we did not observe any significant differences in the HBZ-specific antibody responses among the different clinical subtypes of ATL (data not shown). Although further studies are needed to clarify the reason for this difference, it is possible that both the viral subgroup and host genetic background (including HLA type) determine the anti-HBZ antibody response, since our recent study indicated that different HTLV-1 subgroups are characterized by different patterns of viral and host gene expression in HAM/TSP patients via independent mechanisms of direct transcriptional regulation [36].

In this study, using a sandwich ELISA based on two novel in-house mAbs, we report the absolute quantification of HBZ protein in naturally HTLV-1-infected cells. The amount of endogenous HBZ protein varied between 76.9 and 371.4 ng/mL in HTLV-1-infected T cell lines and between 64.2 and 96.4 ng/mL in the primary PBMCs of ATL patients, which was two- to tenfold less than the amount overexpressed in HBZ-transfected 293T cells. HBZ protein was detected only in a proportion of the PBMCs from acute ATL patients, and was not detected in the PBMCs from HAM/TSP patients or ACs. Our data also indicate that the expression levels of HBZ mRNA were not correlated with HBZ protein levels. However, interestingly, our data show that the amount of HBZ protein in primary HTLV-1-infected cells was correlated with PVL. One possible explanation for the discrepancy in expression levels between HBZ protein and mRNA is that HBZ RNA and protein may have distinct functions. A previous report by Satou et al. showed that a mutant HBZ without the first ATG codon (required for translation) still promoted T cell proliferation but did not suppress HTLV-1 transcription, whereas a missense HBZ mutant that possibly disrupted the RNA secondary structure did not promote T cell proliferation [7]. Namely, the activation and polyclonal proliferation of HTLV-1-infected cells, which is closely related to HAM/TSP pathogenesis, may depend on HBZ RNA structure rather than on HBZ protein. Indeed, we previously reported that HBZ mRNA is constitutively expressed in the PBMCs of all HAM/TSP patients, and that HBZ mRNA expression in HTLV-1-infected cells was decreased after successful immunomodulatory treatment for HAM/TSP [8]. Furthermore, a recent report by Mitobe et al. provided evidence that HBZ RNA associates with genes involved in the cell cycle and cell proliferation and survival, whereas HBZ protein is more closely related to the immunological properties of T cells [37]. It may be possible that the most important actions of HBZ, which are critical to HTLV-1 persistence, are exerted at the RNA level, and not the protein level. Another possibility is that HTLV-1 may have evolved to minimize HBZ translation. Consequently, HBZ protein suppress the translation efficiency of HBZ itself. This hypothesis may be supported by the findings that HBZ is an important target of the T cell response [13, 14] and most HBZ mRNA and protein is retained in the nucleus [38].

Meanwhile, although both HBZ protein expression and immune responses against HBZ were extremely low in vivo, recent studies demonstrated the importance of the in vivo functions of the HBZ protein. Namely, a recent in silico analysis showed that efficient CTL recognition and a strong immune response to HBZ is important for efficient control of viral replication [13], which reduces the risk of HTLV-1-associated diseases in HTLV-1-infected individuals [15, 16]. Also, it has recently been reported that HBZ-targeted vaccination by recombinant vaccinia viruses could induce CTLs, which have an anti-lymphoma effect in mouse and macaque models of ATL, suggesting the potential of HBZ as a target antigen for immunotherapy [18]. These results demonstrated the potentially beneficial roles of HBZ-specific immune responses against HTLV-1-associated diseases and the importance of HBZ protein.

Since transgenic expression of HBZ in CD4+ T cells induced both T-cell lymphoma and systemic inflammation in mice [10], it is clear that HBZ protein is also critical for the development of HTLV-1-associated diseases. Our present data suggest the possibility that the low but distinct expression of HBZ protein in the PBMCs of ATL patients along with the increased number of infected cells may be associated with the development of ATL. Therefore, targeted immunotherapy against HBZ may be effective for the clearance of HTLV-1-infected cells and the prevention of HTLV-1-associated diseases.

## Conclusions

In summary, we demonstrated the absolute quantification of HBZ protein in naturally HTLV-1-infected cells of ATL patients. The very low immunogenicity of HBZ in HTLV-1-infected individuals, which may be due to minimized HBZ protein translation, is a sophisticated viral strategy for evasion from the host T cell response, resulting in the survival of HTLV-1-infected cells and consequent pathogenesis of HTLV-1-related disorders.

## Additional files

10.1186/s12977-016-0263-z **Figure S1:** Amino acid sequence of HBZ. **A.** The amino acid sequence of the HBZ protein is shown. The sequences of the peptides used to generate the anti-HBZ monoclonal antibodies are underlined. **B.** The histidine-tagged recombinant HBZ protein, which was produced by a wheat germ extract based cell free transcription and translation system (WEPRO7240H Expression kit; CellFree Sciences, Japan), was analyzed by SDS-PAGE. Gels were scanned and the levels of HBZ protein were quantified by BIO-RAD Quantity One 4.3.1 software using densities of known concentrations of BSA. **Figure S2:** Development of an ELISA for the detection of HBZ protein in HTLV-1-infected cells. HBZ protein levels in cell lysates were evaluated by in-house sandwich ELISA using mAbs against HBZ (i.e., clone #91-1 for capture and clone #20-H12 for detection). As shown, a 96-well flat-bottom plate was coated with an anti-HBZ mAb (clone #91-1: rat IgG1 mAb raised against peptide #3). Then, a second anti-HBZ mAb (clone #20-H12: mouse IgG1 mAb raised against peptide #2) conjugated to HRP was used as the detection antibody. **Figure S3:** Western blotting analysis of HBZ protein in HTLV-1-infected cell lines using various monoclonal antibodies (mAbs). HBZ protein expression in HTLV-1-infected cell lines was analyzed by western blotting using various anti-HBZ mAbs (i.e. P6-A7, #20-H12, #7-1, #91-1). Clone #91-1 (raised against peptide #3, rat IgG1) showed the highest sensitivity for detecting HBZ protein. Histone H3 was used as a loading control for the nuclear fraction.
10.1186/s12977-016-0263-z **Table S1:** Characterization of the produced monoclonal antibodies against HBZ. **Table S2:** Laboratory findings of HTLV-1 infected individuals for whom PBMCs and plasma samples were measured for HBZ protein and anti-HBZ Abs.

## Abbreviations

HTLV-1: human T-cell leukemia virus type-1
ATL: adult T-cell leukemia
HAM/TSP: HTLV-1-associated myelopathy/tropical spastic paraparesis
ACs: asymptomatic carriers
NCs: normal uninfected healthy controls
PVL: proviral load
HPRT: hypoxanthine phosphoribosyltransferase
