# Supplementary material for: Absolute quantification of HTLV-1 basic leucine zipper factor (HBZ) protein and its plasma antibody in HTLV-1 infected individuals with different clinical status
Source: Retrovirology. 2016 Apr 27;13:29. doi: 10.1186/s12977-016-0263-z (PMC4847349; doi:10.1186/s12977-016-0263-z)
Supplement: Supplementary file 1 — 10.1186/s12977-016-0263-z Figure S1: Amino acid sequence of HBZ. A. The amino acid sequence of the HBZ protein is shown. The sequences of the peptides used to generate the anti-HBZ monoclonal antibodies are underlined. B. The histidine-tagged recombinant HBZ protein, which was produced by a wheat germ extract based cell free transcription and translation system (WEPRO7240H Expression kit; CellFree Sciences, Japan), was analyzed by SDS-PAGE. Gels were scanned and the levels of HBZ protein were quantified by BIO-RAD Quantity One 4.3.1 software using densities of known concentrations of BSA. Figure S2: Development of an ELISA for the detection of HBZ protein in HTLV-1-infected cells. HBZ protein levels in cell lysates were evaluated by in-house sandwich ELISA using mAbs against HBZ (i.e., clone #91-1 for capture and clone #20-H12 for detection). As shown, a 96-well flat-bottom plate was coated with an anti-HBZ mAb (clone #91-1: rat IgG1 mAb raised against peptide #3). Then, a second anti-HBZ mAb (clone #20-H12: mouse IgG1 mAb raised against peptide #2) conjugated to HRP was used as the detection antibody. Figure S3: Western blotting analysis of HBZ protein in HTLV-1-infected cell lines using various monoclonal antibodies (mAbs). HBZ protein expression in HTLV-1-infected cell lines was analyzed by western blotting using various anti-HBZ mAbs (i.e. P6-A7, #20-H12, #7-1, #91-1). Clone #91-1 (raised against peptide #3, rat IgG1) showed the highest sensitivity for detecting HBZ protein. Histone H3 was used as a loading control for the nuclear fraction. [file 12977_2016_263_MOESM1_ESM.pptx]

## Slide 1
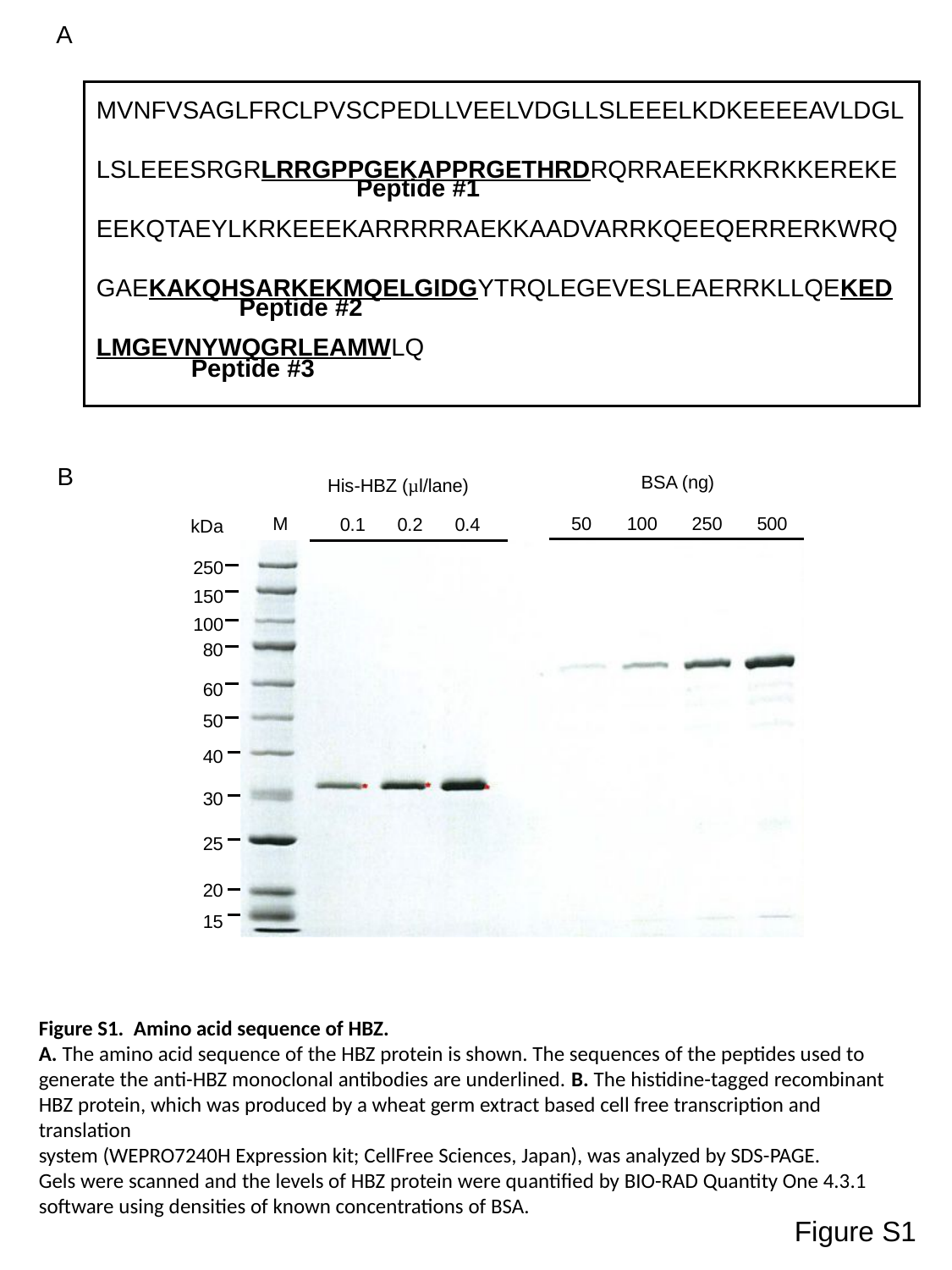

A
MVNFVSAGLFRCLPVSCPEDLLVEELVDGLLSLEEELKDKEEEEAVLDGLLSLEEESRGRLRRGPPGEKAPPRGETHRDRQRRAEEKRKRKKEREKEEEKQTAEYLKRKEEEKARRRRRAEKKAADVARRKQEEQERRERKWRQGAEKAKQHSARKEKMQELGIDGYTRQLEGEVESLEAERRKLLQEKEDLMGEVNYWQGRLEAMWLQ
Peptide #1
Peptide #2
Peptide #3
B
BSA (ng)
His-HBZ (µl/lane)
50
100
250
500
M
0.1
0.2
0.4
kDa
250
150
100
80
60
50
40
30
25
20
15
Figure S1. Amino acid sequence of HBZ.
A. The amino acid sequence of the HBZ protein is shown. The sequences of the peptides used to
generate the anti-HBZ monoclonal antibodies are underlined. B. The histidine-tagged recombinant
HBZ protein, which was produced by a wheat germ extract based cell free transcription and translation
system (WEPRO7240H Expression kit; CellFree Sciences, Japan), was analyzed by SDS-PAGE.
Gels were scanned and the levels of HBZ protein were quantified by BIO-RAD Quantity One 4.3.1
software using densities of known concentrations of BSA.
Figure S1

## Slide 2
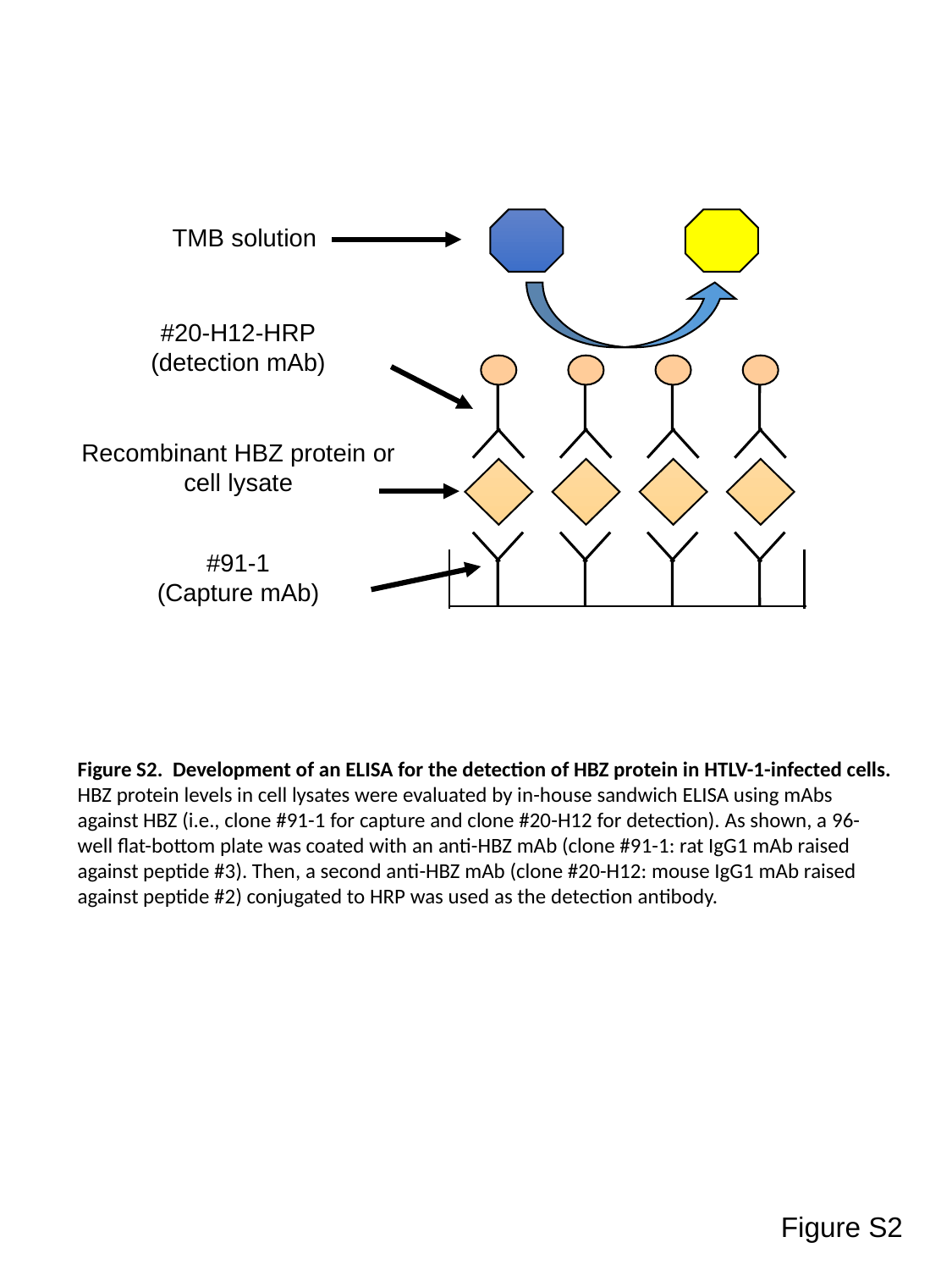

TMB solution
#20-H12-HRP
(detection mAb)
Recombinant HBZ protein or cell lysate
#91-1
(Capture mAb)
Figure S2. Development of an ELISA for the detection of HBZ protein in HTLV-1-infected cells.
HBZ protein levels in cell lysates were evaluated by in-house sandwich ELISA using mAbs against HBZ (i.e., clone #91-1 for capture and clone #20-H12 for detection). As shown, a 96-well flat-bottom plate was coated with an anti-HBZ mAb (clone #91-1: rat IgG1 mAb raised against peptide #3). Then, a second anti-HBZ mAb (clone #20-H12: mouse IgG1 mAb raised against peptide #2) conjugated to HRP was used as the detection antibody.
Figure S2

## Slide 3
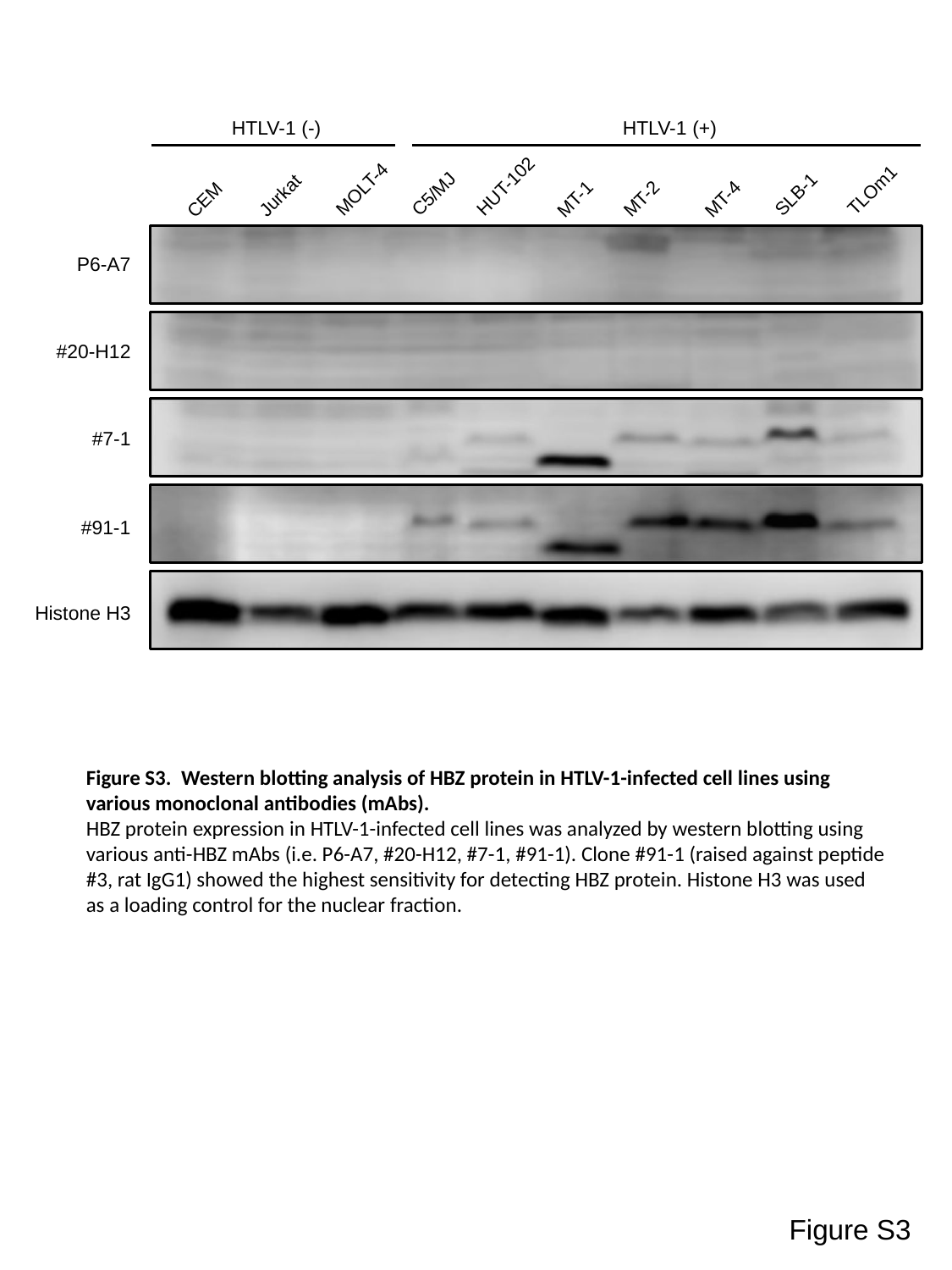

HTLV-1 (-)
HTLV-1 (+)
HUT-102
MOLT-4
TLOm1
C5/MJ
SLB-1
Jurkat
MT-2
MT-4
MT-1
CEM
P6-A7
#20-H12
#7-1
#91-1
Histone H3
Figure S3. Western blotting analysis of HBZ protein in HTLV-1-infected cell lines using various monoclonal antibodies (mAbs).
HBZ protein expression in HTLV-1-infected cell lines was analyzed by western blotting using various anti-HBZ mAbs (i.e. P6-A7, #20-H12, #7-1, #91-1). Clone #91-1 (raised against peptide #3, rat IgG1) showed the highest sensitivity for detecting HBZ protein. Histone H3 was used as a loading control for the nuclear fraction.
Figure S3
