# Supplementary material for: Absolute quantification of HTLV-1 basic leucine zipper factor (HBZ) protein and its plasma antibody in HTLV-1 infected individuals with different clinical status
Source: Retrovirology. 2016 Apr 27;13:29. doi: 10.1186/s12977-016-0263-z (PMC4847349; doi:10.1186/s12977-016-0263-z)
Supplement: Supplementary file 2 — 10.1186/s12977-016-0263-z Table S1: Characterization of the produced monoclonal antibodies against HBZ. Table S2: Laboratory findings of HTLV-1 infected individuals for whom PBMCs and plasma samples were measured for HBZ protein and anti-HBZ Abs. [file 12977_2016_263_MOESM2_ESM.docx]

**Table S1: Characterization of the produced monoclonal antibodies against HBZ**

| **Name of clone** | **Antigen** | **Animal immunized** | **Antibody isotypes** |
| --- | --- | --- | --- |
| P6-A7 | peptide #1 | mouse | IgG2b |
| P4-E12 | peptide #1 | mouse | IgG2b |
| #20-H12 | peptide #2 | mouse | IgG1 |
| #21-1 | peptide #3 | rat | IgG2a |
| #91-1 | peptide #3 | rat | IgG1 |
| #1-1 | Recombinant HBZ | mouse | IgG2a |
| #7-1 | Recombinant HBZ | mouse | IgG2b |

**Table S2: Laboratory findings of HTLV-1 infected individuals for whom PBMCs and plasma samples were measured for HBZ protein and anti-HBZ Abs.**

| Case | Age | Sex | HBZ protein ^a^  (PBMCs) | anti-HBZ Abs ^b^  (Plasma) | HBZ mRNA ^c^  (PBMCs) | HTLV-1 proviral load ^d^  (copies/10^4^PBMCs) |
| --- | --- | --- | --- | --- | --- | --- |
| AC-1 | 60 | M | 9.95 | 0.000 | 0.020 | 233 |
| AC-2 | 51 | F | 13.94 | 0.000 | 0.449 | 1193 |
| AC-3 | 63 | F | 17.92 | 0.021 | 0.086 | 104 |
| AC-4 | 39 | F | 15.76 | 0.004 | 0.145 | 1309 |
| ATL-1 | 66 | F | 23.70 | 0.000 | 3.570 | 6832 |
| ATL-2 | 74 | F | 96.40 | 0.011 | 0.000130 | 23822 |
| ATL-3 | 75 | F | 35.33 | 0.000 | 0.000110 | 19577 |
| ATL-4 | 73 | M | 88.29 | 0.035 | 0.000105 | 12673 |
| ATL-5 | 55 | F | 64.20 | 0.022 | 0.000006 | 14739 |
| HAM-1 | 72 | F | 11.49 | 0.017 | 0.645 | 2426 |
| HAM-2 | 65 | F | 5.98 | 0.004 | 0.102 | 100 |
| HAM-3 | 62 | F | 11.05 | 0.000 | 0.140 | 893 |
| HAM-4 | 59 | F | 5.65 | 0.002 | 0.405 | 206 |
| HAM-5 | 73 | F | 11.65 | 0.000 | 1.380 | 2019 |
| HAM-6 | 66 | F | 17.69 | 0.013 | 0.914 | 1155 |
| HAM-7 | 59 | M | 8.35 | 0.018 | 1.850 | 1078 |
| HAM-8 | 62 | M | 14.63 | 0.030 | 0.169 | 1426 |
| HAM-9 | 42 | M | 14.63 | 0.480 | 0.506 | 1136 |
| HAM-10 | 43 | M | 10.12 | 0.371 | 0.587 | 2142 |

^a^ HBZ protein expression levels in naturally infected PBMCs were evaluated by an in-house sandwich ELISA using mAbs against HBZ.

^b^ Anti-HBZ antibody levels in plasma were determined by ELISA using a recombinant HBZ protein.

^c^ HBZ mRNA levels in PBMCs were determined by real-time PCR. The relative HTLV-1 HBZ mRNA load was calculated by the following formula: HTLV-1 HBZ mRNA load = value of HBZ / value of HPRT (hypoxanthine ribosyl transferase).

^d^ The amount of the HTLV-1 proviral load was calculated using β-actin as an internal control through the following formula: HTLV-1 *tax* copy number per 1 × 10^4^ PBMCs = [(*tax* copy number)/(β-actin copy number/2)] × 10^4^.
